# Reparative and toxicity-reducing effects of liposome-encapsulated saikosaponin in mice with liver fibrosis

**DOI:** 10.1042/BSR20201219

**Published:** 2020-08-13

**Authors:** Li-Yen Shiu, Han Hsiang Huang, Chun Yin Chen, Hsia-Ying Cheng, Chih I. Chen, Shyh Ming Kuo

**Affiliations:** 1 Department of Medical Research, E-Da Hospital, Kaohsiung City, Taiwan; 2 Department of Veterinary Medicine, National Chiayi University, Chiayi City, Taiwan; 3 Department of Biomedical Engineering, I-Shou University, Kaohsiung City, Taiwan; 4 Central for General Education, I-Shou University, Kaohsiung City, Taiwan; 5 Division of Colon and Rectal Surgery, Department of Surgery, E-Da Hospital, Kaohsiung City, Taiwan

**Keywords:** cytotoxicity, liposome, liver fibrosis, Saikosaponin

## Abstract

Saikosaponin d (SSd), a primary active component of the Chinese herb *Bupleurum falcatum*, has antitumor and antiliver fibrosis effects. However, the toxicity of SSd at high doses can induce conditions such as metabolic disorders and hemolysis *in vivo*, thus hampering its clinical use. The present study investigated the toxicity-reducing effects of liposome encapsulation of pure SSd and the therapeutic action of SSd-loaded liposomes (Lipo-SSd) in liver fibrosis *in vitro* and *in vivo*. Lipo-SSd (diameter, 31.7 ± 7.8 nm) was prepared at an entrapment efficiency of 94.1%. After 10-day incubation, a slow release profile of 56% SSd from Lipo-SSd was observed. The IC_50_ of SSd on hepatic stellate cells was approximately 2.9 μM. Lipo-SSd exhibited much lower cytotoxicity than did pure SSd. In the *in vivo* toxicity assay, Lipo-SSd significantly increased mice survival rate and duration compared with pure SSd at the same dose. These *in vitro* and *in vivo* data indicate that liposomal encapsulation can reduce the cytotoxicity of SSd. The histopathological analysis results demonstrated that in mice with thioacetamide-induced liver fibrosis, Lipo-SSd exerted more obvious fibrosis- and inflammation-alleviating and liver tissue-reparative effects than did pure SSd; these effects are potentially attributable to the sustained release of SSd. In conclusion, Lipo-SSd fabricated here have antiliver fibrosis effects and lower toxicity compared with that of pure SSd.

## Introduction

Liver fibrosis, characterized by an accumulation of extracellular matrix in the liver, is generally preceded by chronic inflammation. Moreover, persistence of inflammation in the liver is correlated with progressive liver fibrosis. Activation of liver fibrosis involves two main stages: (1) initiation, related to paracrine-mediated alterations in gene expression, which make cells receptive to cytokines and other stimuli, and (2) perpetuation, the consequence of maintenance of the aforementioned stimuli, which further increases cytokine secretion as well as the progression of extracellular matrix remodeling [1]. In the end stage of fibrosis, nodular transformation of the liver and impedance of portal blood flow and cirrhosis occur [2,3]. During the progression of liver injury, quiescent hepatic stellate cells (HSCs) are subject to activation stimuli and thereafter develop a myofibroblast-like phenotype. These activated HSCs are highly proliferative and synthesize collagen types I and III, α-smooth muscle actin, and extracellular matrix components. Several cytokines and growth factors secreted by activated HSCs are causes of fibrosis. Of these cytokines, TGF-β1 provides the most potent fibrogenesis stimulus in the liver. Moreover, many studies have reported that angiotensin and free radicals (including reactive oxygen species) are involved in liver fibrosis formation: angiotensin, produced by activated HSCs, stimulates reactive oxygen species generation [3]. Moreover, in preliminary human studies, angiotensin inhibitors and antioxidants such as S-adenosyl-L-methionine and vitamin E have been found to exhibit antifibrosis activities.

Chinese herbs represent major constituents of complementary and alternative medicine. Because their use involves natural healing methods and their curative effects are long-lasting, herbal medicines are currently replacing synthetic pharmaceuticals and are regarded as sources of novel bioactive substances [1]. Moreover, some Chinese herbal medicines have antioxidant and immunoregulatory effects and thus exert strong antiliver fibrosis activity [4].

In Chinese pharmacopoeia, Chaihu is documented as a mixture of dried roots of *Bupleurum chinense* DC. and *B. scorzonerifolium* Willd. (Umbelliferae) [5]. Moreover, in Japanese Pharmacopoeia (16th edition), the official botanical origin of Bupleuri Radix (*saiko* in Japanese) is the roots of the Chinese herb *B. falcatum* L. In Japan and Taiwan, *B. falcatum* L. is believed to have therapeutic effects against chronic hepatitis and autoimmune diseases. Saikosaponin d (SSd) is an active and structurally steroid-like saponin in *B. falcatum*. SSd has anti-inflammatory [6,7], antiviral, antifibrosis and immunomodulatory actions, and thus is considered for use in pharmacological applications and in alternative therapy for several disorders [8–11]. In our previous studies, SSd could induce apoptosis in both tumor cells and HSCs through the caspase-3 and mitochondrial pathways, suggesting that SSd potentially has antitumor and antiliver fibrosis capacity and further strengthening its potential for biomedical and therapeutic applications [12–14].

Liposomes, which have a colloidal and vesicular structure, are composed of one or more lipid bilayers [15]. Liposomes have been widely used as carriers in the drug delivery system for carrying agents with poor bioavailability and poor water solubility, including some proteins, peptides, antibiotics, and anticancer agents. Liposomes are biocompatible and biodegradable and have structures similar to those of cell membranes. Thus, liposomes have become a suitable and popular alternative for drug vehicles for improving the pharmacodynamics or therapeutic effects of drugs [16,17]. Among the liposome classes, phosphatidylcholine liposomes are the most widely used because their phospholipid components are similar to the relevant components of cell membranes. We used the phospholipid 1,2-distearoyl-sn-glycero-3-phosphocholine (DSPC) for liposome preparation in our previous studies, where we fabricated DSPC liposome-encapsulated astaxanthin to treat alcohol-induced liver fibrosis. The results demonstrated that our astaxanthin liposomes have protective and therapeutic potential, thus demonstrating that the established liposome encapsulation can increase the bioavailability and potency of astaxanthin *in vivo*.

In the present study, to reduce the toxicity of SSd at high doses, we prepared SSd-loaded liposomes (Lipo-SSd) and then evaluated their reparative efficacy in mice with thioacetamide (TAA)-induced liver fibrosis through various analytical methods.

## Materials and methods

### Chemicals and animals

SSd was purchased from Chem Faces (U.S.A.). TAA was purchased from Sigma. Cholesterol, DSPC (molecular weight = 790.15 Da), chloroform, and methanol were obtained from Sigma (St. Louis, MO, U.S.A.). All chemicals used in the present study were of reagent grade.

The animal research was approved by the Institutional Animal Care and Use Committee of I-Shou University, Taiwan (AUP-106-50-01). In total, 90 wild-type C57BL/6J male mice (6 weeks old) were used in our mouse model of liver fibrosis.

### Preparation and characterization of *Lipo-SSd*

**Figure 1:**
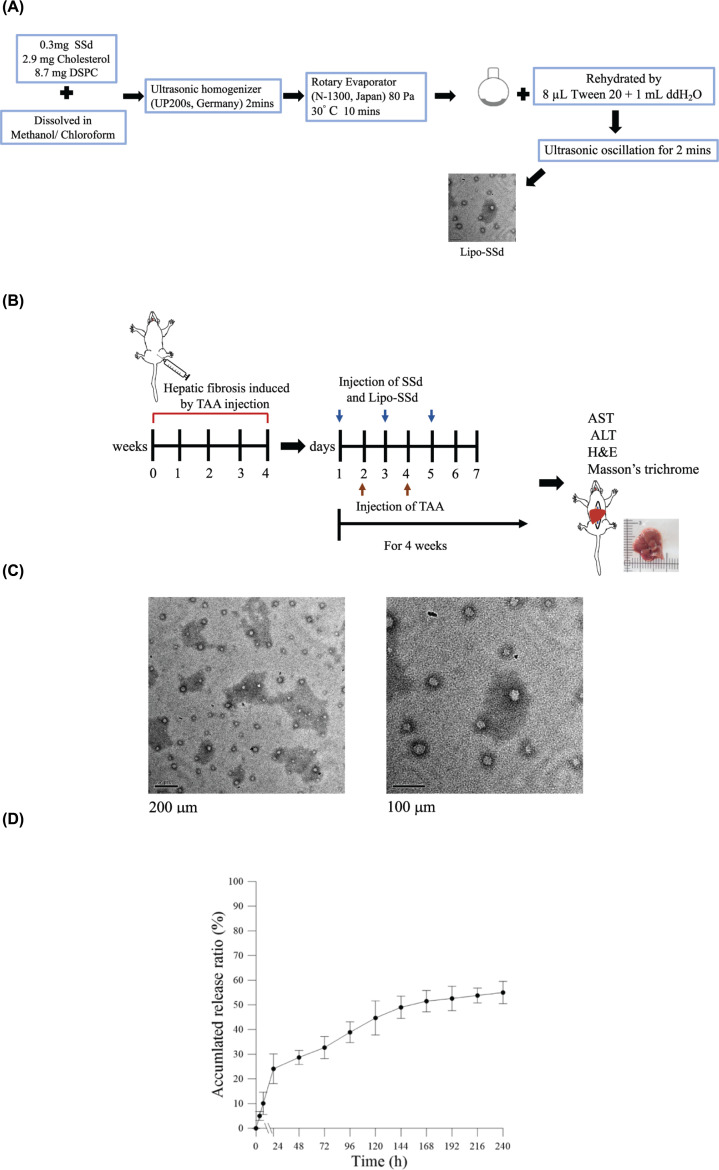
Preparation and characterization of *Lipo-SSd* (**A**) Schematic of the Lipo-SSd preparation process. (**B**) Schematic representation and time scale of TAA-induced liver fibrosis establishment and treatments using pure SSd or Lipo-SSd in mice. (**C**) Transmission electron microscopy images of Lipo-SSd. (**D**) SSd release profile of Lipo-SSd.

Lipo-SSd were prepared using the evaporation sonication method with some modification [18]. In brief, liposomes were first prepared by dissolving the phospholipid DSPC (8.7 mg), cholesterol (2.9 mg), and SSd (0.3 mg) in methanol–chloroform (1:3, v/v). This mixture was then homogenized for 120 s (UP 200S, Germany) and dried in a rotary evaporator (N-1300, Japan) into a thin film. This film was rehydrated with deionized water (1 ml) and subjected to sonication for 2 min (Figure 1A). The size of Lipo-SSd was calculated through random sampling of approximately 50 individual liposomes by using a transmission electron microscope [19]. Entrapment efficiency (EE) of Lipo-SSd was determined as follows: 1 ml of the solution containing the prepared Lipo-SSd was centrifuged at 10,000 rpm for 10 min, and then the amount of nonencapsulated SSd in the supernatant was measured through high-performance liquid chromatography (HPLC; Agilent 1100 series). A standard concentration curve of SSd was established for determining the amount of SSd encapsulated in liposomes. EE was finally calculated as [(total amount of SSd − amount of nonencapsulated SSd)/total amount of SSd] × 100%.

The *in vitro* release of SSd from Lipo-SSd was also assessed. In total, 100 µl of Lipo-SSd was added to a 1.5-ml microcentrifuge tube. The tube was then placed on a shaker set at 37°C and 40 rpm. At particular timepoints, the sample was centrifuged at 10,000 rpm for 10 min. The amount of nonencapsulated SSd in the supernatant was determined through HPLC. Independent experiments were preformed three times in triplicate. The *in vitro* release rate was calculated as [(total amount of SSd − amount of residual SSd)/total amount of SSd] × 100%.

### Cell culture and treatment with SSd and Lipo-SSd

Human HSC cell line LX-2, purchased from Merck Millipore (SCC064), was seeded in 96-well plates at a density of 5000 cells/well in Dulbecco’s modified Eagle’s medium (Gibco) supplemented with 100 U/ml penicillin, 100 µg/ml streptomycin, and 10% fetal bovine serum (Gibco). After 24 h of incubation at a temperature of 37°C and 5% CO_2_ concentration, the medium was replaced with fresh medium containing various concentrations of pure SSd or Lipo-SSd. The viability of HSCs was examined through 3-(4,5-dimethylthiazol-2-yl)-2,5-diphenyltetrazolium bromide (MTT) assay. In brief, after the aforementioned 24-h treatment, 20 μl of MTT solution was added to the cells before incubation for 3 h. The formazan precipitate was dissolved in 100 μl of dimethyl sulfoxide, and the absorbance was measured at 570 nm on a multiplate reader (Thermo Scientific, Waltham, MA, U.S.A.). Cell viability was also examined using a live/dead cell assay (Invitrogen, Carlsbad, CA, U.S.A.). Briefly, 1 ml of PBS solution containing 2.5 μl/ml of 4 μM ethidium homodimer-1 (EthD-1) assay solution and 1 μl/ml of 2 μM calcein AM solution was prepared according to the instructions. The assay solution (100 μl) was added to the culture, and the mixture was placed at 37°C in 5% CO_2_ for 15 min. The upper fluid was removed, and the sample was observed using a fluorescence microscope at excitation filters of 494 nm (green, calcein) and 528 nm (red, EthD-1) (Olympus IX71, Japan). The green fluorescence in each experimental group was further quantified by Image-J software (version 1.50; National Institutes of Health, U.S.A.) to compare the amounts of live cells of the respective group.

### Apoptosis analysis

HSCs were seeded in 12-well plates at a density of 1 × 10^5^ cells/well and incubated for 24 h at 37°C. The cultured medium was replaced with fresh medium, and various concentrations of pure SSd or Lipo-SSd were added to the well before incubation for 24 h. Apoptosis assay for HSCs was performed using a staining kit containing FITC-conjugated annexin V/PI (BD Biosciences, San Diego, CA, U.S.A.) according to the manufacturer’s instructions. In brief, the cells were detached from the culture flask through trypsinization and washed with phosphate-buffered saline. Cell pellets were suspended in 1× binding buffer and stained with 5 μl of annexin V-FITC and 10 μl of PI for 15 min. The cells were then subjected to fluorescence-activated cell sorting analysis using a flow cytometer (BD Accuri C6).

### Animal experiment design

A TAA working solution (80 mg/ml) was prepared in phosphate-buffered saline. The mice were administered three doses per week of 100 μl of this TAA solution through intraperitoneal injection for 4 weeks. The mice were randomized into the experimental group (with TAA-induced liver fibrosis; *n*=18) and the control group (with no liver fibrosis; *n*=3). The experimental mice received continual intraperitoneal injection of TAA (2 doses/week) to ensure the occurrence of liver injury after SSd treatment initiation.

The mice were divided into three groups: TAA-induced fibrotic liver (no SSd treatment), SSd (three injections of 20 mg/kg pure SSd for 4 weeks), and Lipo-SSd (three injections of 20 mg/kg Lipo-SSd for 4 weeks). The *in vivo* experiments were performed over the course of 8 weeks. In the first 4 weeks, TAA-induced liver fibrosis was allowed to develop, and the subsequent 4 weeks consisted of treatment with intraperitoneal injection of 100 μl of SSd or Lipo-SSd (once on day 1 and then three times per week thereafter).

In all experiments, all mice were anesthetized through intraperitoneal injection of tiletamine + zolazepam (40 mg/kg) and xylazine (10 mg/kg). At the end of the experiments, mice were killed through CO_2_ overdose. Blood and liver samples were harvested for serum biochemical and histopathological analyses. Figure 1B presents a schematic representation and timepoints of the establishment of TAA-induced liver injury and its subsequent treatment in the mice.

### Histopathological analysis

After the 4-week treatment with pure SSd or Lipo-SSd, the livers of the mice were collected, and the liver tissues were fixed in 10% neutral-buffered formalin. The liver samples were then dehydrated in graded ethanol solutions, cleared in xylene, embedded in paraffin blocks, and cut into 3-µm–thick sections. Hematoxylin and eosin (H&E) staining was used for histopathological examination of the sections. Masson trichrome staining was also applied to assess collagen content in the livers of the mice treated with SSd or Lipo-SSd. ImageJ software was used to measure the collagen content in each group [20]. In brief, the color settings in ImageJ remained constant through the analysis of the blue-stained areas in the samples. Samples were evaluated at 100× magnification, and the calculation was repeated in four microscopic fields.

### Western blot

Western blot analysis was conducted based on the following procedures: 10% sodium dodecyl sulfate-polyacrylamide gel electrophoresis was prepared to separate the protein samples. Polyvinylidene difluoride membrane was prewetted in methanol for a few seconds and then in transfer buffer for 30 min. The proteins of the samples were transferred and blotted onto the membrane. The membranes were incubated in 5% skim milk powder in Tris-buffered saline with 0.05% Tween 20 for 1 h at room temperature to block nonspecific protein binding. The membrane was then incubated with primary anti-mouse antibodies of procaspase-3, α-SMA and GAPDH overnight at 4°C.

### Serum alanine aminotransferase and aspartate aminotransferase measurement

Alanine aminotransferase (ALT) and aspartate aminotransferase (AST) assays were applied to evaluate the progression or recovery from TAA-induced liver fibrosis and damage after treatment with SSd or Lipo-SSd. Serum samples for the ALT and AST assays were prepared according to the manufacturer’s instructions (AAT Bioquest, CA, U.S.A.). In brief, each 100-μl sample was mixed with the provided ALT or AST enzyme on a plate and incubated at 37°C for 20–30 min. Absorbance was measured at 575 nm on a microplate reader.

### Survival assays

Two animal survival assays were performed to evaluate the *in vivo* toxicity of pure SSd and Lipo-SSd, one of which was intraperitoneally injected into the mice at 20–50 mg/kg; their survival for the subsequent 7 days after injection was analyzed.

Another survival assay was conducted using only two doses of SSd and Lipo-SSd (20 and 40 mg/kg) administered intraperitoneally three times per week. The survival of the mice was analyzed for the subsequent 4 weeks.

### Statistical analysis

The data are presented as means ± standard deviations. The experimental data were pooled from three independent experiments. The results were analyzed through one-way analysis of variance with SPSS (version 17.0) for the existence of significant differences (with *P*<0.05) between the control and experimental groups.

## Results and discussion

### Encapsulation and release of SSd from Lipo-SSd

The Lipo-SSd preparation procedure is illustrated in Figure 1A. Transmission electron microscopy revealed that the prepared Lipo-SSd were spherical and 31.7 ± 7.8 nm in diameter (Figure 1C). Their EE was 94.1%, approximately 56% of the SSd released from Lipo-SSd after 10 days of incubation (Figure 1D).

#### SSd and Lipo-SSd cytotoxicity assay

**Figure 2:**
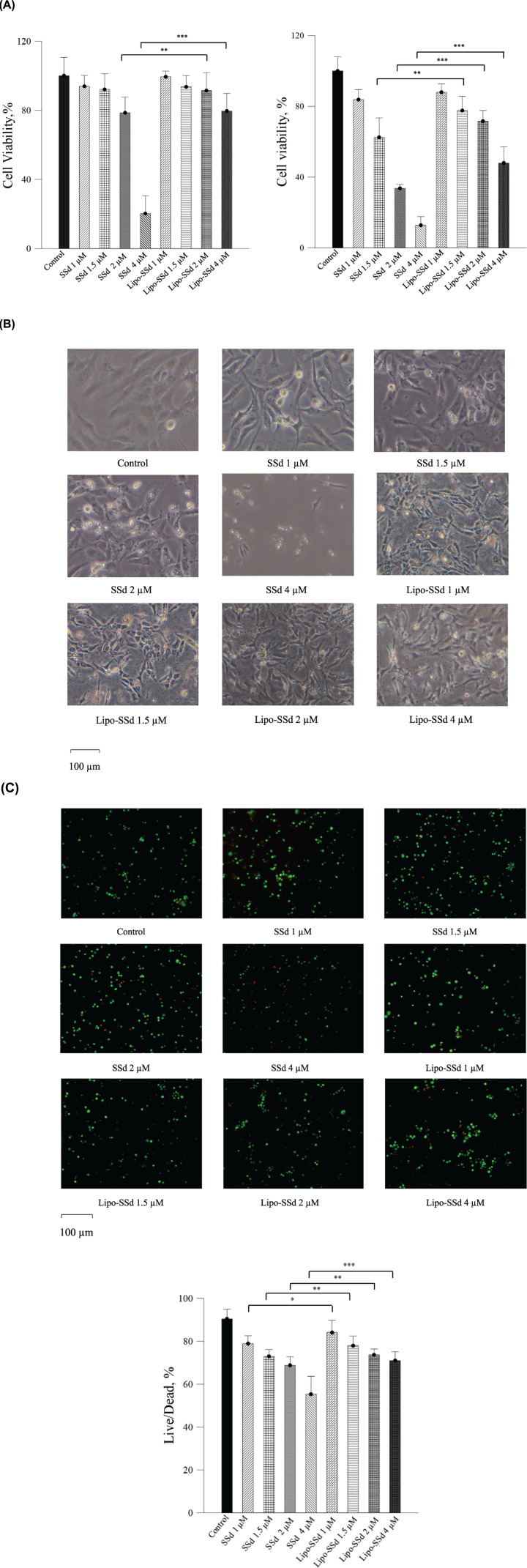
SSd and Lipo-SSd cytotoxicity assay (**A**) Results of cell viability analysis of different SSd and Lipo-SSd on HSCs through MTT assay after 24 and 48 h of incubation. (**B**) Impact on HSC morphology monitored through light microscopy (magnification, 100×) after 24 h of incubation. (**C**) Live/dead staining images of HSCs treated with SSd or Lipo-SSd after 24 h of incubation. The live/dead cell rate of each group was calculated and statistically analyzed with one-way ANOVA (*P<0.5, **P<0.01, and ***P<0.001).

The cytotoxic effects of SSd and Lipo-SSd on HSCs were assessed using MTT assay. HSCs were exposed to SSd or Lipo-SSd for 24 h. HSC viability was >79% upon exposure to 1–2 µM SSd but decreased considerably to approximately 20% upon exposure to 4 µM SSd. Therefore, for HSCs, the IC_50_ of SSd was approximately 2.9 µM. By contrast, Lipo-SSd caused lower cytotoxicity in HSCs than did SSd. Upon exposure to 1–4 µM Lipo-SSd, HSC viability was >80%, indicating that liposomal encapsulation could reduce the cytotoxicity of SSd *in vitro* (Figure 2A). The results of cell morphological analysis demonstrated that HSCs gradually shrunk and their pseudopods shortened upon exposure to 2 µM SSd. Upon exposure to 4 µM SSd, more HSCs shrunk, rounded up, and detached from the culture plate. By contrast, HSC morphology was unaffected by any of the Lipo-SSd treatments, even after 24 h of incubation (Figure 2B). Live/dead cell staining demonstrated that the number of dead HSCs increased with the increase in SSd concentration from 1 to 4 µM, with the greatest number of dead HSCs noted after exposure to 4 µM Lipo-SSd. By contrast, no changes in the number of dead HSCs were observed in relation to the concentration of Lipo-SSd (Figure 2C). These results corroborated the MTT assay results that liposomal encapsulation can reduce SSd cytotoxicity *in vitro*.

**Figure 3:**
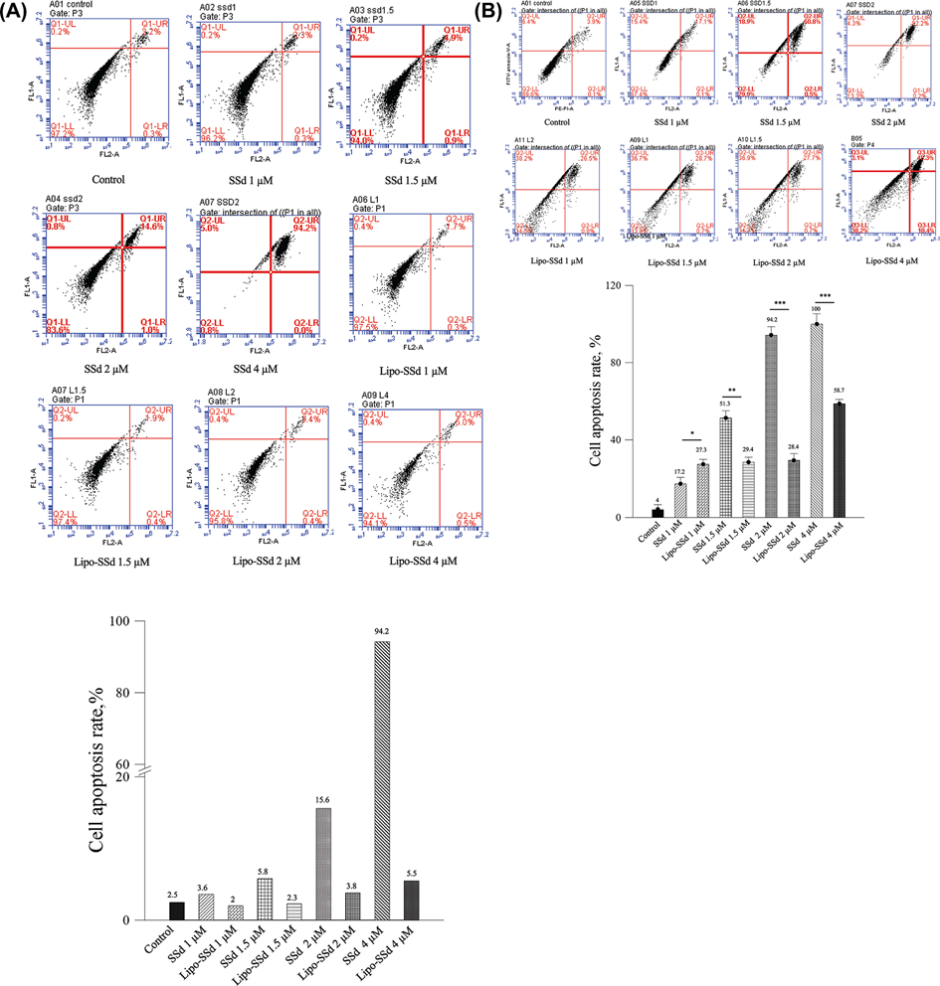
Apoptosis assay of HSCs after treatment with different concentrations of SSd and Lipo-SSd Apoptosis assay of HSCs after treatment with different concentrations of SSd and Lipo-SSd after (**A**) 24 h and (**B**) 48 h of incubation (*P<0.5, **P<0.01, and ***P<0.001).

**Figure 4:**
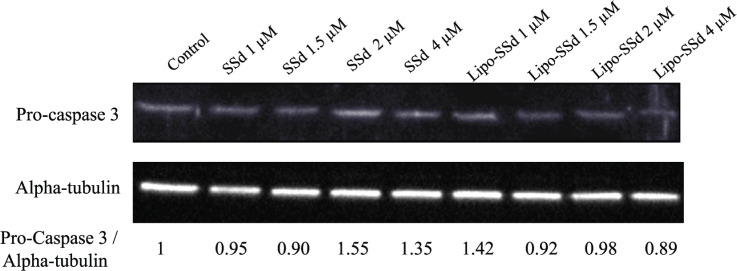
Western blot of Pro-Caspase 3 levels in HSCs Western blot of Pro-Caspase 3 levels in HSCs after treatment with different concentrations of SSd or Lipo-SSd.

SSd can induce apoptosis in both tumor cells and HSCs [12–14]. In the present study, 2 µM SSd led to an approximately 4.33-fold higher apoptosis rate in HSCs compared with that observed for 1 µM SSd, but the cell survival rate remained at 84.4% (Figure 3). Notably, SSd cytotoxicity significantly decreased after liposomal encapsulation at the same concentration. The apoptosis rate for 4 µM SSd was considerably decreased from 94.2% to 5.5% after liposomal encapsulation. The *in vitro* cytotoxicity assay results thus demonstrated that the toxicity of SSd at a high dose could be reduced through application of the liposomal encapsulation technique. Moreover, Western blot results confirmed that pure SSd may induce apoptosis through procaspase-3–related pathways (Figure 4).

### Liposome-encapsulation of SSd increased the survival rate

**Figure 5:**
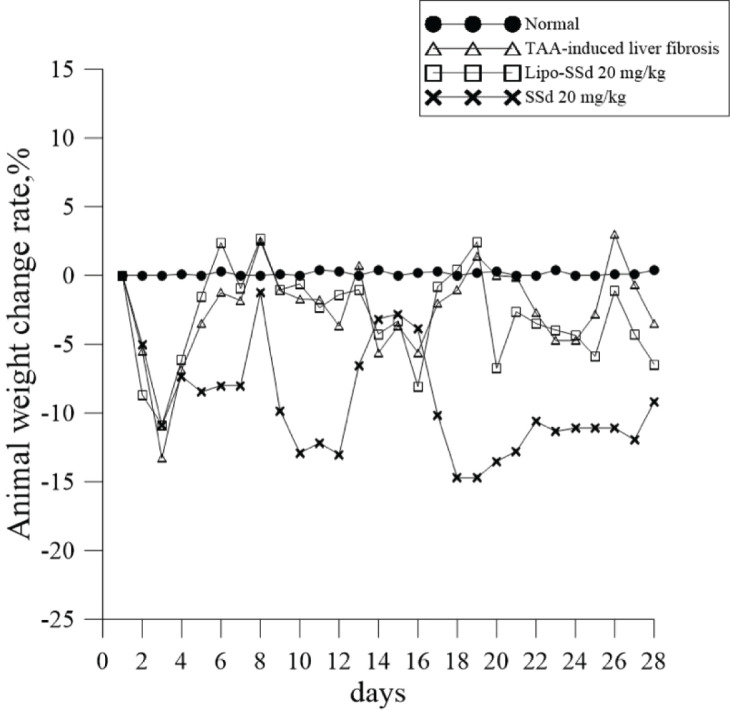
Liposome-encapsulation of SSd increased the survival rate Lipo-SSd demonstrating lower weight decrease (within 10%) than SSd in mice with TAA-induced liver fibrosis. SSd reduced mouse weight by nearly 15% during days 10–13 and 18–27 of treatment.

SSd has strong hemolytic activity because of its α-hydroxyl function at C16 [21]. Two *in vivo* toxicity assays were performed to evaluate the toxicity of SSd and toxicity-alleviating capacity of Lipo-SSd. A 10–50-fold higher SSd dose was administered to our mice with TAA-induced liver fibrosis compared with the doses used in previous studies [8,22]. The results indicated that the administration of Lipo-SSd could lead to increased survival rate and duration compared with those following the administration of SSd at the same dose. The survival rate in the mice was <50% and the survival duration was <1 week after treatment with ≥25 mg/kg pure SSd, the survival rate was 100% and survival duration was ≥7 days after treatment with 20–50 mg/kg Lipo-SSd. Pure liposomes did not cause any toxicity in the mice (Table 1). In more injections of SSd or Lipo-SSd survival experiments, the 2-week survival rate was 100% for mice receiving a dose of 20 mg/kg Lipo-SSd and mice all survived in the designed 4-week duration. The 2-week survival rate decreased to 20% for mice receiving higher dose of 20 mg/kg Lipo-SSd, and the average survival period also decreased. However, mice receiving pure SSd all demonstrated much lower survival rate. The mice all died after injection of 40 mg/kg pure SSd (Table 2). Our *in vivo* results thus corroborate our *in vitro* results, demonstrating that liposomal encapsulation can reduce SSd cytotoxicity. Moreover, the average weight of each mouse in the experimental group further reflected and confirmed that liposome encapsulation could reduce SSd toxicity *in vivo* because 20 mg/kg SSd led to a 10% greater reduction in mouse body weight compared with 20 mg/kg Lipo-SSd (Figure 5).

**Table 1:** Survival of mice 7 days after one intraperitoneal injection of SSd or Lipo-SSd

Dose (mg/kg)	Survival rate (%)	Survival to 7-d (number)	Average survival period (Day)	Animal number (*n*)
Injection of SSd				
20	72.7	7	5.5	11
25	63.6	7	5.1	11
50	15.4	2	1.07	13
Injection of Lipo-SSd				
20	100	5	7	5
25	100	5	7	5
50	100	3	7	5
Injection of pure liposome				
100	100	3	7	3

More mice were tested in the SSd group than in the Lipo-SSd group to verify SSd toxicity.

**Table 2:** Survival of mice 4 weeks after three intraperitoneal injections of SSd or Lipo-SSd

Dose (mg/kg)	2-week survival rate (%)	Survival to 4-week (number)	Average survival period (week)
Injection of SSd			
20	40	4	2
40	0	0	1
Injection of Lipo-SSd			
20	100	10	4
40	20	2	2

*n*=10 for each group.

### Lipo-SSd improved liver healing

**Figure 6:**
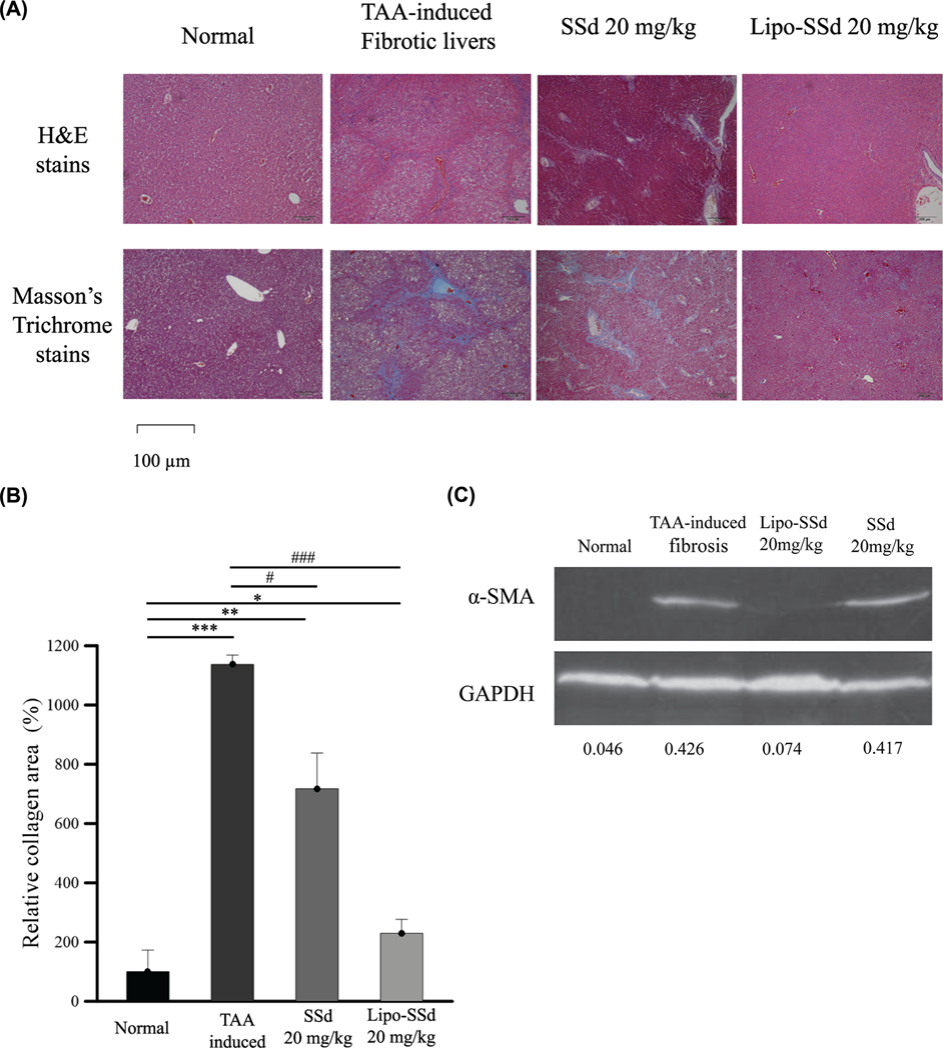
Lipo-SSd improved liver healing (**A**) Results of H&E staining and Masson trichrome staining of the sections of experimental mouse livers, which received treatment with SSd or Lipo-SSd (magnification, 100×). (**B**) Collagen content in the livers of mice in each group, semi-quantitated using ImageJ software. (**C**) Western blot of α-SMA levels of the experimental mouse livers. Western blot for α-SMA and GAPDH was run on identical gel and the blot in each lane was separated by black lines. *P<0.5, **P<0.01, and ***P<0.001, all compared with the healthy liver group; ^#^*P*<0.5 and ^###^*P*<0.001, all compared with the untreated TAA-induced liver fibrosis group.

TAA, CCl_4_, dimethylnitrosamine, and diethylnitrosamine are commonly used hepatotoxins for inducing hepatotoxicity, chronic liver injury, liver necrosis, liver tumor formation, and liver fibrosis in rodent models. Pericentral and periportal fibrosis is noted in TAA-induced liver injury; TAA represents quicker emergence of portal–central and portal–portal fibrotic septa than does CCl_4_ [23–25]_._ The results of histopathological analysis using Masson trichrome staining and H&E staining demonstrated that at 20 mg/kg, both pure SSd and Lipo-SSd led to decrease in fibrotic tissue–collagen content compared with no treatment (Figure 6A). In healthy mouse livers, hepatic sinusoids were found near the hepatocytes at 100× magnification (Figure 6A). In the livers of mice with untreated TAA-induced liver fibrosis, infiltration of inflammatory cells in several areas, hepatocyte necrosis and degeneration, and increased amounts of a collagen-like substance were observed, thus confirming the induction of liver necrosis and fibrosis. Moreover, the results of Masson trichrome staining demonstrated that the blue-stained areas mainly appeared close to central veins, revealing that the TAA-induced liver fibrosis was pericentral and periportal, which is consistent with the previous findings [26–29]. The livers of mice treated with 20 mg/kg pure SSd exhibited less necrosis and moderately fewer collagen-like areas, but hepatocyte degeneration and infiltration of inflammatory cells were still observed (Figure 6A). H&E staining results supported those of Masson trichrome staining. In mice treated with Lipo-SSd, H&E staining revealed noticeably fewer inflammatory areas, decreased hepatocyte degeneration and necrosis, and fewer collagen-like areas in the liver sections. The collagen quantitative results were consistent with the histopathological findings. The semiquantitative data from Masson trichrome staining demonstrated that at 20 mg/kg, both pure SSd and Lipo-SSd significantly reduced the collagen content in the liver compared with no treatment. Notably, the results of the histopathological analysis and collagen content assay indicated that the fibrotic livers of mice treated with 20 mg/kg Lipo-SSd healed to achieve a healthy status. By contrast, 20 mg/kg pure SSd treatment reduced the liver collagen content by approximately 39% compared with no treatment. Therefore, low-dose Lipo-SSd can significantly ameliorate TTA-induced liver fibrosis in mice but pure SSd, even at the same dose, cannot (Figure 6B). The protein level α-SMA in the TAA-induced liver fibrosis treated with or without SSd and Lipo-SSd was assessed by Western Blot in the murine model *in vivo* (Figure 6C). As a result, administration with 20 mg/kg Lipo-SSd largely decreased the proteins of α-SMA compared with that of TAA-induced fibrosis without treatment (positive control). About 20 mg/kg SSd did not show clearly decrease in α-SMA compared to positive control. The Western Blot data further verified the histopathologic H&E and Masson Trichrome staining findings, which showed the strongest inhibition in collagen formation in 20 mg/kg Lipo-SSd administered group. The *in vivo* histopathological, biochemistry and Western Blot data demonstrated that Lipo-SSd is able to efficiently and stably ameliorate fibrosis on the murine TAA-induced liver injury/fibrosis model.

**Figure 7:**
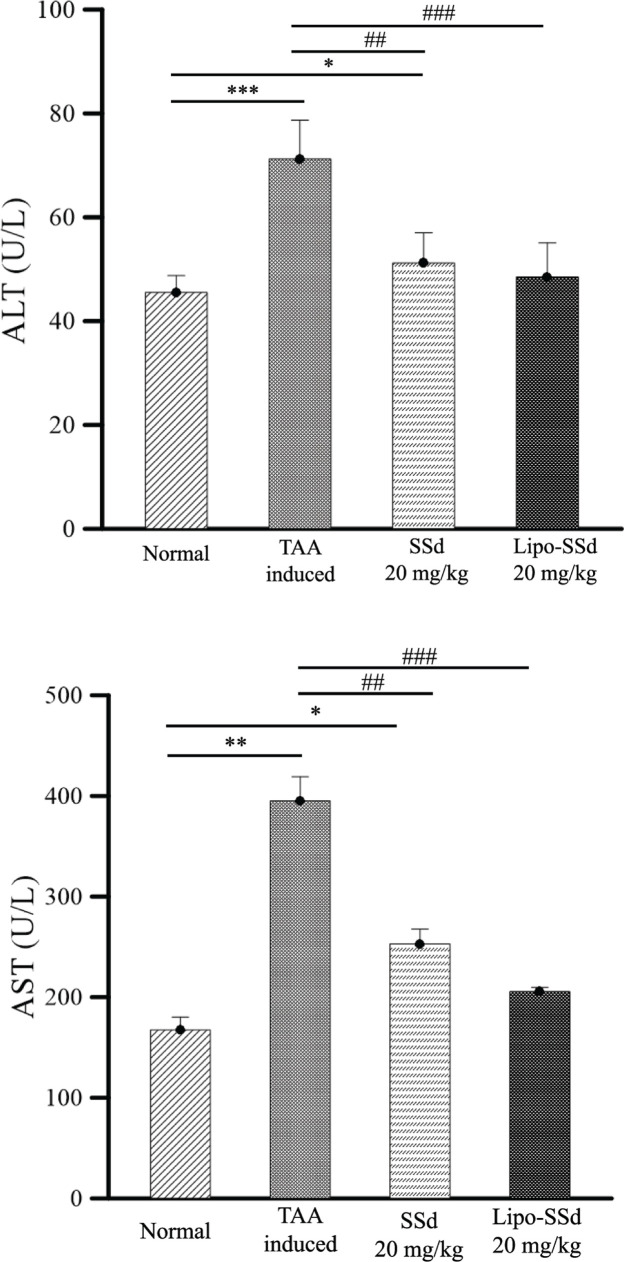
Effects of SSd and Lipo-SSd on ALT and AST levels in mice with TAA-induced liver fibrosis Both Lipo-SSd and pure SSd significantly reduced ALT and AST levels, but Lipo-SSd and SSd did not exhibit differences in liver function–improving effects. *P<0.5, **P<0.01, and ***P<0.001, all compared with the healthy liver group; ^##^*P*<0.01, and ^###^*P*<0.001, all compared with the untreated TAA-induced liver fibrosis group.

Serum ALT and AST levels were further examined to validate the liver function of mice in each experimental group. The results revealed that ALT levels after pure SSd and Lipo-SSd treatment at 20 mg/kg returned to a healthy range, indicating that intraperitoneal injection of Lipo-SSd and SSd could recover liver function. In particular, Lipo-SSd significantly reduced ALT and AST levels compared with levels for pure SSd treatment and no treatment, approaching healthy levels (Figure 7). The ALT and AST levels in the group treated with Lipo-SSd and that treated with pure SSd were generally consistent with our histopathological analysis findings. Intraperitoneal injection of Lipo-SSd or pure SSd at 20 mg/kg significantly reduced AST and ALT levels compared with no treatment, thus verifying that both Lipo-SSd and pure SSd administration can improve liver function in mice with TAA-induced liver fibrosis.

A study reported that SSd from *B. falcatum* can induce mammalian HSC apoptosis through several mechanisms, such as caspase 3-dependent, caspase 3-independent, and mitochondrial pathways [12]. We previously reported that the encapsulation or aggregation of nanoparticles can enhance and strengthen the apoptotic pathways induced by natural marine compounds in bladder cancer cells [30]. Thus, in the present study, we investigated the reparative and toxicity-reducing effects of Lipo-SSd in liver fibrosis. Although an increase in pro-caspase 3 level was not observed in some Lipo-SSd–treated groups, it was observed in most pure SSd–treated groups, suggesting that caspase 3 is involved in apoptosis induction by SSd. Decreases in pro-caspase 3 levels were observed in most groups treated with Lipo-SSd, whereas increases pro-caspase 3 levels were found in some groups treated with pure SSd, suggesting that caspase-3 plays a critical role in apoptosis induced by Lipo-SSd. This result can be explained by the nanoparticle encapsulation results: the encapsulated nanoparticles could promote the apoptotic pathways of antitumor compounds in tumor cells [30]; therefore, liposome encapsulation may similarly improve the antifibrosis actions, specifically through caspase 3-related pathways in HSCs, but these results warrants further investigation.

Taken together, Lipo-SSd caused lower cytotoxicity than did pure SSd in HSCs *in vitro* and in the TAA liver fibrosis mice model; moreover, exposure to Lipo-SSd increased survival duration and rates, even compared with 10- to 50-fold higher doses of pure SSd. These results verify that liposome encapsulation can minimize cytotoxicity and toxicity *in vitro* and *in vivo*. Moreover, although pure SSd also induced more apoptosis in HSCs than did Lipo-SSd, pure SSd had greater cytotoxicity than Lipo-SSd in HSCs at identical doses. The histopathological analysis results revealed that Lipo-SSd had greater liver fibrosis–reducing, liver-restorative, and inflammation-alleviating effects than did pure SSd at identical doses in mice with TAA-induced liver fibrosis. AST and ALT data further validated the activities of Lipo-SSd and SSd in improving liver function in mice with TAA-induced liver fibrosis. Western blot results also suggested that liposome encapsulation aids in specifically directing SSd-induced apoptosis through caspase-3–related pathways. Moreover, liposome encapsulation could reduce SSd toxicity, and Lipo-SSd could more effectively ameliorate liver fibrosis than pure SSd at the same dose, suggesting that liposome encapsulation is a practicable modification for the therapeutic use of SSd because it can simultaneously enhance its pharmacological effects and reduce its toxicity *in vivo*.

## Abbreviations

ALT: alanine aminotransferase
AST: aspartate aminotransferase
DSPC: 1,2-distearoyl-sn-glycero-3-phosphocholine
H&E: hematoxylin and eosin
HSC: hepatic stellate cell
MTT: 3-(4,5-dimethylthiazol-2-yl)-2,5-diphenyltetrazolium bromide
